# Supplementary material for: Transcriptomic Analyses of Camellia oleifera ‘Huaxin’ Leaf Reveal Candidate Genes Related to Long-Term Cold Stress
Source: Int J Mol Sci. 2020 Jan 28;21(3):846. doi: 10.3390/ijms21030846 (PMC7037897; doi:10.3390/ijms21030846)
Supplement: Supplementary file 1 [file ijms-21-00846-s001.zip › Supplementary file/Additional file 1-table S1.docx]

Additional file 1: Table S1

**Table S1** Overview of the sequencing and assembly

| Sample  ID | Clean  reads | Base  Number | GC Content | %≥Q30 | Mapped  reads | Mapped  Ratio |
| --- | --- | --- | --- | --- | --- | --- |
| A1 | 21,799,314 | 6,534,692,402 | 46.36% | 92.97% | 16,259,464 | 74.59% |
| A2 | 21,760,919 | 6,518,781,098 | 44.98% | 92.67% | 15,463,973 | 71.06% |
| A3 | 22,443,817 | 6,710,679,034 | 44.44% | 92.56% | 15,808,114 | 70.43% |
| B1 | 21,760,771 | 6,503,059,964 | 44.51% | 93.50% | 15,403,495 | 70.79% |
| B2 | 21,574,878 | 6,456,079,242 | 44.63% | 93.31% | 15,348,394 | 71.14% |
| B3 | 21,905,676 | 6,552,048,682 | 45.10% | 92.97% | 15,666,600 | 71.52% |
| C1 | 21,828,489 | 6,535,669,890 | 44.98% | 92.97% | 15,669,484 | 71.78% |
| C2 | 20,206,690 | 6,042,264,412 | 45.84% | 93.13% | 14,591,526 | 72.21% |
| C3 | 21,607,094 | 6,460,263,978 | 44.82% | 92.96% | 15,274,635 | 70.69% |
