# Supplementary material for: Transcriptomic Analyses of Camellia oleifera ‘Huaxin’ Leaf Reveal Candidate Genes Related to Long-Term Cold Stress
Source: Int J Mol Sci. 2020 Jan 28;21(3):846. doi: 10.3390/ijms21030846 (PMC7037897; doi:10.3390/ijms21030846)
Supplement: Supplementary file 1 [file ijms-21-00846-s001.zip › Supplementary file/Additional file 9-table S9.docx]

**Table S9** Primers of qRT-PCR of the 12 DEGs and one reference gene

| Gene number | Primer name | Primer sequence (5'to3') | TM |
| --- | --- | --- | --- |
| 1 | c122737.graph_c0-1-F | TCCTTCACCTTCAGTATT | 58 |
|  | c122737.graph_c0-1-R | ACTACAGAATCATCAATGG |  |
| 2 | c140493.graph_c0-1-F | CAGCAACCTTCTTGACAG | 58 |
|  | c140493.graph_c0-1-R | TACCTCCACTTCCTTCTTAC |  |
| 3 | c118580.graph_c0-1-F | GATTGTTTGAGAGGCTTTA | 58 |
|  | c118580.graph_c0-1-R | CGAATGTTATGAAGGAAGT |  |
| 4 | c135205.graph_c1-1-F | ACCACAGGACCAAACATC | 58 |
|  | c135205.graph_c1-1-R | GAACGCCAGCATACATTG |  |
| 5 | c125403.graph_c2-1-F | ATAGCACCAAGGAGCATA | 58 |
|  | c125403.graph_c2-1-R | ATGATGAAGGCGATGAAG |  |
| 6 | c137063.graph_c0-1-F | ATAATCTCCTCTCCTACCTT | 58 |
|  | c137063.graph_c0-1-R | TCTCAGTGAATGGCAATG |  |
| 7 | c143173.graph_c0-1-F | AGCCTCTCACTGTTATGC | 58 |
|  | c143173.graph_c0-1-R | ACTATTCCTATCATTCCACCAT |  |
| 8 | c139678.graph_c1-1-F | CGAGAGTGGTGAGTAGAT | 58 |
|  | c139678.graph_c1-1-R | TTAACGGAATATACGCCTTAG |  |
| 9 | c139290.graph_c0-1-F | GTGATTGAATGGTGAAGGTAT | 58 |
|  | c139290.graph_c0-1-R | CACTGCTGTTGATTAGGC |  |
| 10 | c126487.graph_c3-1-F | CCTAATGCCCTGATTGTC | 58 |
|  | c126487.graph_c3-1-R | ATTCTTGTTGTCTCCTTGTT |  |
| 11 | c135177.graph_c0-1-F | TCCTTGCCATCACCATCT | 58 |
|  | c135177.graph_c0-1-R | GAGCCAGTTGCCGAATAC |  |
| 12 | c133783.graph_c0-1-F | GCTTCTTTGACATAGGAG | 58 |
|  | c133783.graph_c0-1-R | ATTTACGGACCATTTGAC |  |
| 13 | ACTIN-1-F | GGTATGCGATGAATAGGA | 58 |
|  | ACTIN-1-R | CAAGGATGGTAGTCTCAA |  |
