# Supplementary material for: Transcriptomic Analyses of Camellia oleifera ‘Huaxin’ Leaf Reveal Candidate Genes Related to Long-Term Cold Stress
Source: Int J Mol Sci. 2020 Jan 28;21(3):846. doi: 10.3390/ijms21030846 (PMC7037897; doi:10.3390/ijms21030846)
Supplement: Supplementary file 1 [file ijms-21-00846-s001.zip › Supplementary file/Additional file 2-figure S1-2.doc]

**Transcriptomic analysis of *Camellia oleifera* ‘huaxin’ Reveals Candidate Genes Related to long-term cold stress during flowering period**

**Lingli Wu^1,2^, Xiaofeng Tan^1,2*^, Jian’an Li^1,2*^, Heping Cao^3^ and Mingwang Shi^4^**

^1^*Key Laboratory of Cultivation and Protection for Non-wood Forest Trees of Ministry of Education and the Key Laboratory of Non-Wood Forest Products of Forestry Ministry, Central South University of Forestry and Technology, Changsha 410004, China*

^2^*Engineering Technology Research Center of Southern Hilly and Mountainous Ecological Non-Wood Forest Industry of Hunan Province*

*^3^U.S. Department of Agriculture, Agricultural Research Service, Southern Regional Research Center, New Orleans, Louisiana 70124, USA*

*^4^ Henan Institute of Science and Technology, Xinxiang, Henan 453003, China*

**^*^**For correspondence: tanxiaofengcn@126.com; [lja0731@126.com](mailto:lja0731@126.com)

**Additional file 1 : figure S1**

**A_B**

**A_C**

**B_C**

**Figure S1. Enrichment scatter diagram of differential expression gene KEGG pathway.** Each circle in the figure represents a KEGG pathway, the ordinate represents the name of the pathway, and the abscissa represents the enrichment factor, which represents the ratio of the gene proportion annotated to a pathway in the differential gene to that annotated to the pathway in all genes. The larger the enrichment factor is, the more significant the enrichment level of differentially expressed genes in this pathway is. The circle color represents Q-value, Q-value is the p value corrected by multiple hypothesis test, the smaller Q-value is, the more reliable the enrichment significance of differential expression genes in this pathway is. The size of the circle indicates the number of genes enriched in the pathways, and the larger the circle, the more genes.

**Figure S2. Effect of different temperature treatments on galactose content in *C. oleifera* 'Huanxin' leaves.** A: 25°C; B: 6°C; C: low environmental temperature. Data represent the mean ± SE (n = 3). Lowercase letters indicate significant differences among different temperature treatments at *P* ≤ 0.05 according to Duncan’s multiple range test (DMRT).
